# Cytogenetics, Typification, Molecular Phylogeny and Biogeography of *Bentinckia* (Arecoideae, Arecaceae), an Unplaced Indian Endemic Palm from Areceae

**DOI:** 10.3390/biology12020233

**Published:** 2023-02-01

**Authors:** Suhas K. Kadam, Rohit N. Mane, Asif S. Tamboli, Sandip K. Gavade, Pradip V. Deshmukh, Manoj M. Lekhak, Yeon-Sik Choo, Jae Hong Pak

**Affiliations:** 1 Research Institute for Dok-do and Ulleung-do Island, Department of Biology, School of Life Sciences, Kyungpook National University, Daegu 41566, Republic of Korea; 2 Angiosperm Taxonomy Laboratory, Department of Botany, Shivaji University, Kolhapur 416004, India; 3 Department of Botany, Rayat Shikshan Sanstha’s, Balwant College, Vita 415311, Sangli, India; 4 Department of Botany, Shri Swami Vivekanand Shikshan Santha’s, Dattajirao Kadam Arts, Science and Commerce College, Ichalkaranji 416115, Maharashtra, India

**Keywords:** Arecaceae, *Bentinckia*, biogeographic analysis, karyomorphology, molecular phylogeny, typification

## Abstract

**Simple Summary:**

*Bentinckia* is an Indian endemic genus belonging to the tribe Areceae (Arecaceae). This genus contains two species, *B. condapanna* and *B. nicobarica*, and both need to be conserved as they come under the threatened category. *Bentinckia*, along with nine genera, remains unplaced in Areceae. The members of the unplaced Areceae show characteristics corresponding to all the subtribes. Therefore, morphologically it is difficult to assign any subtribes to these genera. Many molecular phylogenetic analyses have reported the relationships within Areceae. However, all of these are unable to show confident position and support for the species. In the present article, we constructed the molecular phylogeny of Areceae based on an appropriate combination of chloroplast and nuclear loci that satisfactorily depicts the phylogenetic positions of all species from Areceae. Phylogeny and evolutionary history disclose that *Bentinckia* together with unplaced *Clinostigma* and *Cyrostachys* show a close relationship with the subtribe Arecinae and might have originated in Eurasia and India. In addition, this study reports a taxonomic revision of *Bentinckia*. In addition, it provides a new chromosome number (cytotype), i.e. 2*n* = 30 for *B. condapanna*. This study will form the very basis for assessing and refining the systematic position of all the species from the tribe Areceae.

**Abstract:**

*Bentinckia* is a genus of flowering plants which is an unplaced member of the tribe Areceae (Arecaceae). Two species are recognized in the genus, viz. *B. condapanna* Berry ex Roxb. from the Western Ghats, India, and *B. nicobarica* (Kurz) Becc. from the Nicobar Islands. This work constitutes taxonomic revision, cytogenetics, molecular phylogeny, and biogeography of the Indian endemic palm genus *Bentinckia*. The present study discusses the ecology, morphology, taxonomic history, distribution, conservation status, and uses of *Bentinckia*. A neotype was designated for the name *B. condapanna*. Cytogenetical studies revealed a new cytotype of *B. condapanna* representing 2*n* = 30 chromosomes. Although many phylogenetic reports of the tribe Areceae are available, the relationship within the tribe is still ambiguous. To resolve this, we carried out Bayesian Inference (BI) and Maximum Likelihood (ML) analysis using an appropriate combination of chloroplast and nuclear DNA regions. The same phylogeny was used to study the evolutionary history of Areceae. Phylogenetic analysis revealed that *Bentinckia* forms a clade with other unplaced members, *Clinostigma* and *Cyrostachys*, and together they show a sister relationship with the subtribe Arecinae. Biogeographic analysis shows *Bentinckia* might have originated in Eurasia and India.

## 1. Introduction

Arecaceae (Palmae), a monocotyledonous family, is classified into five subfamilies, namely Arecoideae Burnett, Coryphoideae Griff., Ceroxyloideae Drude, Calamoideae Griff., and Nypoideae Griff. Arecoideae is the largest and the most diverse subfamily [1]. Approximately 60% of the palm genera (107 out of 188) and more than 50% of species (ca. 1300 out of 2585) belong to this group. The members of Arecoideae are distributed throughout the tropics and subtropics, occurring mainly in rainforests and to a lesser extent in some seasonally dry habitats. Some species belonging to Arecoideae have economic importance, for example, oil palm, coconut, betel nut, and peach palm; many species are cultivated as ornamental. 

The Areceae is the largest tribe among the palms considering the genera and species number [1]. Areceae represents the following characters: diminutive to robust, acaulescent, erect or rarely climbing, unarmed or armed; pinnate leaves or entire bifid, the leaflet tips entire; sheaths forming a crownshaft or crownshaft absent; infrafoliar or interfoliar inflorescences, spicate to highly branched; inflorescence bracts usually but not always comprising a prophyll and a single peduncular bract; flowers borne in triads at the base, paired or solitary staminate flowers or pits in sunk; pistillate flowers with petals distinct or connate basally, valvate distally; staminodes distinct, or very rarely connate in a conspicuous ring; pseudomonomerous gynoecium; fruit remains with basal or apical stigmatic; epicarp smooth [1]. Areceae consists of 11 subtribes, 61 genera and ca. 660 species. Among these 61 genera, 10 genera with ca. 150 species have not been placed in any subtribes. These species show representative characters of Areceae and have little anatomical diversity, and their general level of variations corresponds to place them in all the subtribes [2]. These unplaced Areceae genera include monotypic *Dransfieldia*, *Dictyosperma*, *Loxococcus* and polytypic *Rhopaloblaste*, *Iguanura*, *Hydriastele*, *Cyrtostachys*, *Bentinckia*, *Clinostigma*, and *Heterospathe*. Although consisting of a considerable number of species, morphological diversity within Areceae is limited, and considerable uncertainty of the phylogenetic relationships among the tribe exists. Few Areceae members can be described as massive trees, whereas many species of the genera *Iguanura* and *Pinanga*, etc., are qualified as ‘palmlets’ of the forest understorey [1].

**Figure 1:**
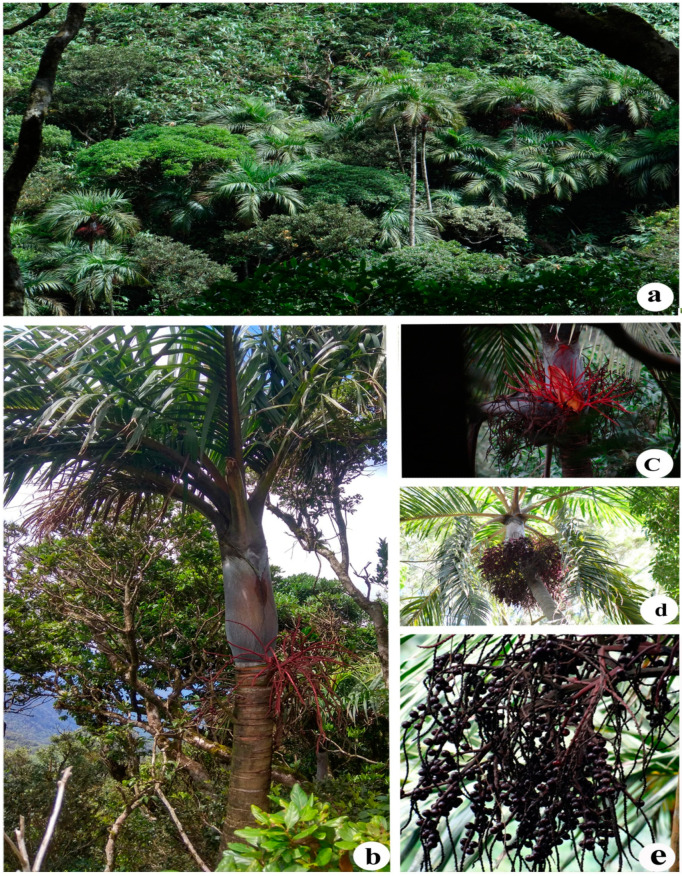
*Bentinckia condapanna* (**a**) habitat, (**b**) habit, (**c**) inflorescence, (**d**) infructescence, and (**e**) fruits.

**Figure 2:**
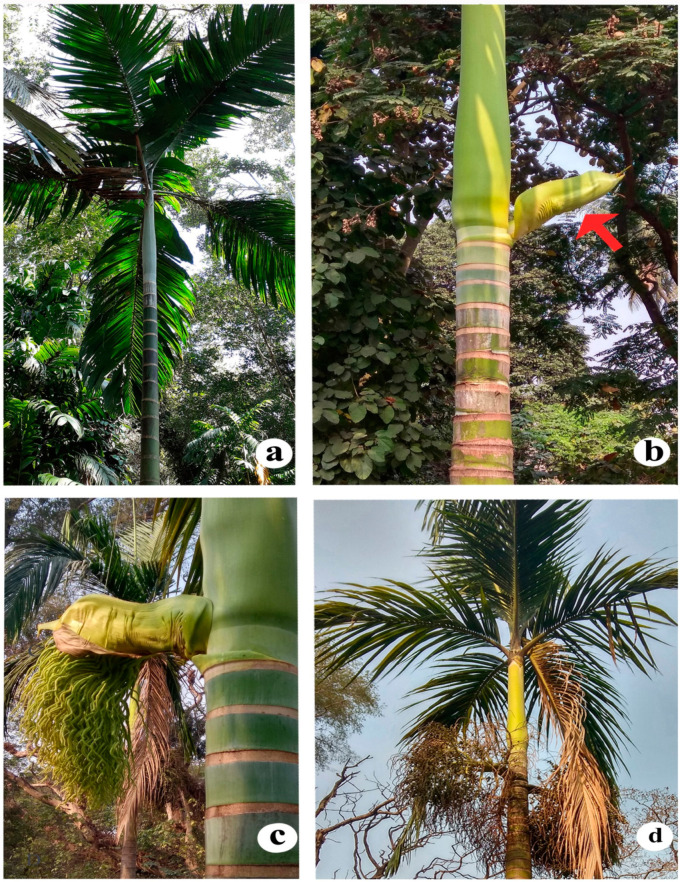
*Bentinckia nicobarica* (**a**) habit, (**b**) spathe, (**c**) inflorescence, and (**d**) infructescence.

*Bentinckia* Berry ex Roxb. is a small genus characterised by its distinct crownshafts and much-branched inflorescence with rachillae bearing flowers in laterally compressed pits [1]. It is endemic to India and comprises only two species, viz. *B. condapanna* (Figure 1) and *B. nicobarica* (Figure 2) [3] and shows disjunct distribution [4]. *Bentinckia condapanna* is only found on mountain cliffs of the southernmost Western Ghats at 1200–1900 m in the forests of Kerala and Tamil Nadu. It is a strong light demander, fog resilient, fire and drought loving, and a good colonizer. It is a very sensitive species in its regeneration process and prefers to grow in open places. The species occur well in shallow, porous soil with good drainage in first or second order streams where a continuous soil moisture regime is ensured frequent precipitation [5]. This species is medicinally important and used in the Siddha system of medicine [6]. Its terminal buds and young leaves are edible. It is cultivated as an ornamental palm in botanical gardens and parks due to its slender stem and feather-like leaves [5]. *Bentinckia nicobarica* grows at low altitudes in the humid forests of the Kamorta, Katchal, Great Nicobar, Nancowry, and Trinket Islands [3,7]. It has a small number of populations in the Nicobar group of islands and is at risk of extinction [8]. 

The generic name *Bentinckia* was validly published in Roxburghs’ Flora Indica [9] with one species, *B. condapanna*. The name was given after Lord William Henry Cavendish Bentick (1774–1839), who was the Governor General of India between 1828 and 1835. The second species of the genus was first described as *Orania nicobarica* Kurz in the Journal of Botany, British and Foreign [10]. Beccari [11] transferred *O. nicobarica* to the genus *Bentinckia*, i.e. *B. nicobarica* (Kurz) Becc.

*Bentinckia condapanna* has brightly coloured fruits and a floral axis. Although its new population grows drastically by seeds, these species are rarely reported from accessible forests as these are cut down for terminal shoots that native people and wild elephants eat. Deforestation for tea plantations is a reason for localised habitat on cliffs of mountains. Habitat loss and overuse are significant threats to the survival of *B. condapanna*. Its Populations in the Western Ghats are decreasing and are much restricted in distribution [12]. *B. condapanna* is particular in its habitat (on cliffs of hills) and thereby restricted in distribution. The distribution is restricted to Kerala and Tamil Nadu, including Agastyamala, Pachakkanaum, Kulathupuzha, Uppupara, Peerumedu, Peppara, Moozhiar, South Travancore, and Tirunelveli [12,13]. Basu et al. [5] reported the distribution in Tirunelveli and Travancore Hills. They examined the habitations of the species in Kalakad Mundanthurai Tiger Reserve (KMTR) (Tamil Nadu) using GPS, Gr5IS, and stratified random sampling techniques. Further, the authors studied the growth habit, silvicultural characters, ethnobotany, places of endemism, the phytogeography parameters of field, and its phytosociological layout. Based on these outcomes, the authors concluded that *B. condapanna* is an endangered species that needs to be conserved immediately. 

*Bentinckia nicobarica* grows with other palms such as *Areca triandra*, *A. catechu*, *Rhopaloblaste augusta*, and *Pinanga manii* at low altitudes in moist forests of Katchal Island. *B. nicobarica* was declared as a threatened species in its natural habitat [14]. The leading causes are habitat alteration, human intervention, expansion of agriculture, annual burning, cutting, and the depletion of natural resources. Due to restricted distribution and probable habitat loss, this palm is categorized as an endangered species in the wild population. There is an urgent need to develop some means of protecting it. 

Cytogenetical data have considerable significance in plant taxonomy, and many researchers have studied the cytogenetics of palms. Although recent information on the comparative cytogenetics of the Arecaceae remains limited, no other large family of tropical woody angiosperms has been studied in better detail. To date, chromosome numbers have been published for approximately 330 species in 126 genera [1]. Chromosome morphology and genome size have been studied in only a few of these species. All reliable chromosome counts clearly show that diploid chromosome numbers in palms range from 2*n* = 26–606 (*Chamaedorea pumila* 2*n* = 26 and *Voanioala gerardii* 2*n* = 606) [1]. Supernumerary chromosomes have also been recorded in *Chelyocarpus*, *Chamaerops*, *Trachycarpus*, *Pritchardia*, and *Desmoncus* [15,16,17]. Chromosome numbers are typically uniform within genera and sometimes within larger groups. However, there are well-documented cases of chromosome number variations within *Phoenix*, *Chamaedorea*, *Ravenea*, and *Dypsis.* In the subfamily Arecoideae, chromosome counts span the entire range of palm diploid chromosome numbers (2*n* = 26–606); however, 2*n* = 32 appears to be the most common number. Although the subfamily Arecoideae is large and morphologically diverse, many studies typically reported 2*n* = 32 chromosome numbers. Arecoideae chromosomes were dominantly metacentric to submetacentric. Sharma [18] and Read [19] reported 2*n* = 32 chromosomes in *Bentinckia condapanna* and *B. nicobarica*, respectively. 

In addition, even after being the oldest monocots to appear in fossil records around 95 million years ago, the relationship within the tribe Areceae is ambiguous [1]. Numerous molecular studies on Areceae have been reported; however, all these reports have been unable to obtain well-resolved molecular phylogeny [1,2]. Moreover, the main focus of these studies has been classification at the family and subfamily level and not at the tribe level of Areceae. Hence, the relationships within the subtribes are poorly established and the monophyly of some of the subtribes remains uncertain. In addition, the position of unplaced members of Areceae has also remained obscure. The basic morphological variation and anatomical diversity among unplaced Areceae correspond to all the subtribes. Therefore, incorporating these species into any particular subtribe is a challenging task. Strong morphological and molecular phylogenetic support is necessary to assign the subtribes to unplaced Areceae. Considering all these aspects, it is clear that there is a need to construct a well-resolved molecular phylogeny of Areceae to resolve taxonomic issues.

The current paper provides taxonomic revision, cytogenetics, and molecular phylogeny of *Bentinckia*. Molecular phylogeny of Areceae is reported based on PRK, RPB2, *acc*D, *rpo*C1, *rbc*L, *rps*16, *trn*L-F, *mat*K, and *ndh*F regions. The phylogenetic position and biogeography of unplaced members of Areceae are also discussed.

## 2. Materials and Methods

### 2.1. Taxon Sampling

**Figure 3:**
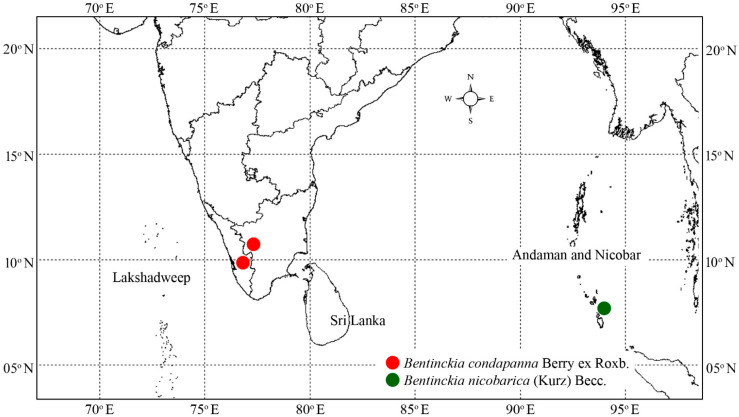
Distribution map of *Bentinckia condapanna* and *B. nicobarica*.

Specimens for both species of *Bentinckia*, viz. *B. condapanna* and *B. nicobarica*, were collected from the Kerala and Kolkata Botanical Gardens, India, respectively. Data for species such as *Arenga wightii* Griff., *A. pinnata* (Wurmb) Merr., *Trachycarpus takil* Becc., and *Hyphaene dichotoma* (D.White bis ex Nimmo) Furtado. were adapted from our previous study [20]. The collection locality of *Bentinckia* is shown in Figure 3. The voucher specimens were collected and submitted to SUK (Department of Botany, Shivaji University, Kolhapur, Maharashtra, India). Herbarium preparation of all specimens followed the protocol reported by Jain and Rao [21]. Plant identification was based on consultation of the relevant literature [3] and the description provided in protologue and type specimens. We constructed a dataset of 67 taxa representing 63 species of the tribe Areceae and 4 of Coryphoideae (outgroup) (Supplementary File S1). This dataset was created by combining sequences produced in the present study and extracted sequences from the NCBI database. The created dataset covers the sampling of all the subtribes and unplaced members of the tribe Areceae.

### 2.2. Cytogenetics

A wild population of sampled *Bentinckia* (fruits and seeds) was used for cytological study. The chromosome preparation was carried out using methods described by Mane and Yadav [22]. Well-spread chromosomes were photographed using a Leica DM 2000. Ten plates of well-segregated metaphase chromosomes were assigned for karyotype analysis as described by Levan et al. [23]. Chromosome morphology was determined by the centromeric index as: short arm × 100/total length of the chromosome. Homologous chromosomes were paired by centromeric index and length. The length of a chromosome was estimated from the mean of the total length of the chromosome. Mean chromosome length (MCL) and the sum of lengths of all chromosomes of the complement (THL) were also calculated. Comparative karyograms were prepared for both species. The degree of karyotype asymmetry was determined following the categories of Stebbins [24] and parameters proposed by Peruzzi & Eroglu [25], viz. the Coefficient of Variation of Chromosome Length (CV_CL_), Coefficient of Variation of Centromeric Index (CV_CI_) and Mean Centromeric Asymmetry (M_CA_).

### 2.3. DNA Extraction, PCR, and Sequencing

DNA from the fresh green leaves of *Bentinckia condapanna* and *B. nicobarica* was extracted using a modified CTAB method reported by Paterson et al. [26]. The PCR amplification of *mat*K, *ndh*F, *rbc*L, RPB2, *rps*16, and PRK genes was carried out as mentioned in a previous study [20]. The sequencing of amplified loci were carried out at Macrogen, Inc. (Republic of Korea). The obtained accession numbers of *acc*D, *mat*K, PRK, *ndh*F, *rbc*L, RPB2, *rpo*C1, *rps*16, and *trn*L-F gene sequences used in the construction of Areceae phylogeny are mentioned in Table S1.

### 2.4. Phylogenetic Analysis

The sequence analysis was done using Sequencher v. 5.1. Multiple sequence alignment of individual genes was carried out in MEGA 11 [27] using the MUSCLE [28] program. All the alignments were refined using a Gblocks server [29]. 

We used ML and BI methods to construct phylogenetic trees of combined (nrDNA + cpDNA) datasets. The jModelTest 2 program [30] was used to select best-fit nucleotide substitution models under AIC. The suggested best-fit model (TVM + I+G) was not present in the MrBayes. Hence, we chose the second model GTR + I+G for the construction of phylogenies. BI phylogeny was constructed in MrBayes v.3.2.7 [31] with similar parameters described in our previous study [20]. ML analysis was also carried out based on the same best-fit model using the IQ tree via IQ tree web server [32]. The number of bootstrap replications was kept at 1000 replicates to assess the robustness of the nodes.

### 2.5. Biogeographic Analysis

Biogeographic areas were defined considering the earlier biogeographic studies and distribution of all Areceae species [33,34]. The distribution range of Areceae was coded as follows: (A) Eurasia up to Wallace’s Line and the Andaman and Nicobar Islands, (B) India and Sri Lanka, (C) Indian Ocean Islands and Madagascar, and (D) the Pacific (areas east of Wallace’s Line and Australia). The S-DIVA analysis was performed on an all-compatible Bayesian tree in RASP v 4.2 [35]. To obtain trustworthy results of biogeographic analysis, 1332 binary trees were used to run S-DIVA. 

## 3. Results

### 3.1. Cytogenetics

#### 3.1.1. Bentinckia Condapanna Berry Ex Roxb

**Figure 4:**
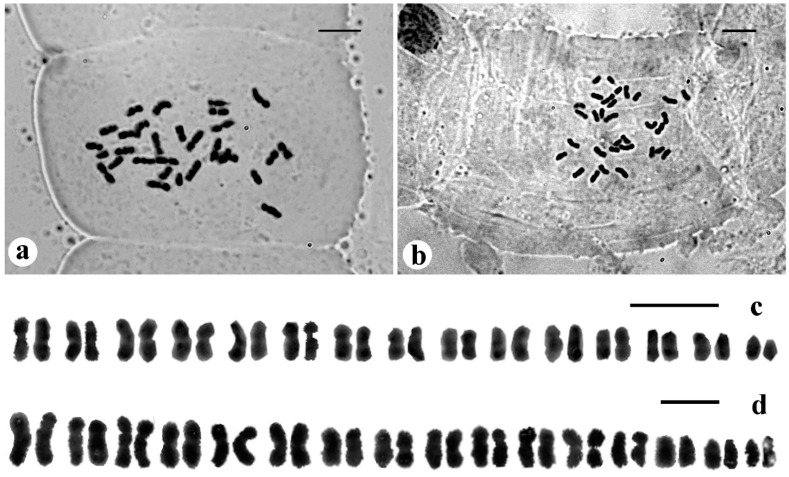
Mitotic metaphase chromosomes and karyograms. (**a**) and (**c**) show *Bentinckia condapanna* (2*n* = 30); (**b**) and (**d**) show *B. nicobarica* (2*n* = 32). Scale bars = 5 μm.

Our study showed that *B. condapanna* collected from Chemunjii, Thiruvananthapuram, Kerala had 2*n* = 30 chromosomes (Figure 4a) (Table 1). This report of *B. condapanna* with 2*n* = 30 chromosomes forms a new cytotype for the species. The CV_CL_ and CV_CI_ were observed at 18.18 and 8.05, respectively. The length of the shortest chromosome was 1.48 μm and the longest chromosome was 3.00 μm. Haploid chromosome length was 34.04 μm. The karyotype formula of this species consisted of 15 median pairs. The karyotype of this species was classified as Stebbins 4B asymmetry class. M_CA_ was 14.05. The karyogram is depicted in Figure 4c.

#### 3.1.2. *Bentinckia Nicobarica* (Kurz) Becc

Our study showed that *B. nicobarica* had a diploid chromosome number of 2*n* = 32 (Figure 4b) (Table 1). The CV_CL_ and CV_CI_ were observed at 27.17 and 11.17, respectively. The shortest chromosome length was 0.91 μm, and the longest chromosome was 2.30 μm in length. Haploid chromosome length was 25.83 μm. The karyotype formula of this species consisted of nine median pairs and seven submedian pairs. The karyotype of this species was classified as Stebbins 3B symmetry class. M_CA_ was 22.02. The karyogram is depicted in Figure 4d.

### 3.2. Molecular Phylogeny of Areceae

**Figure 5:**
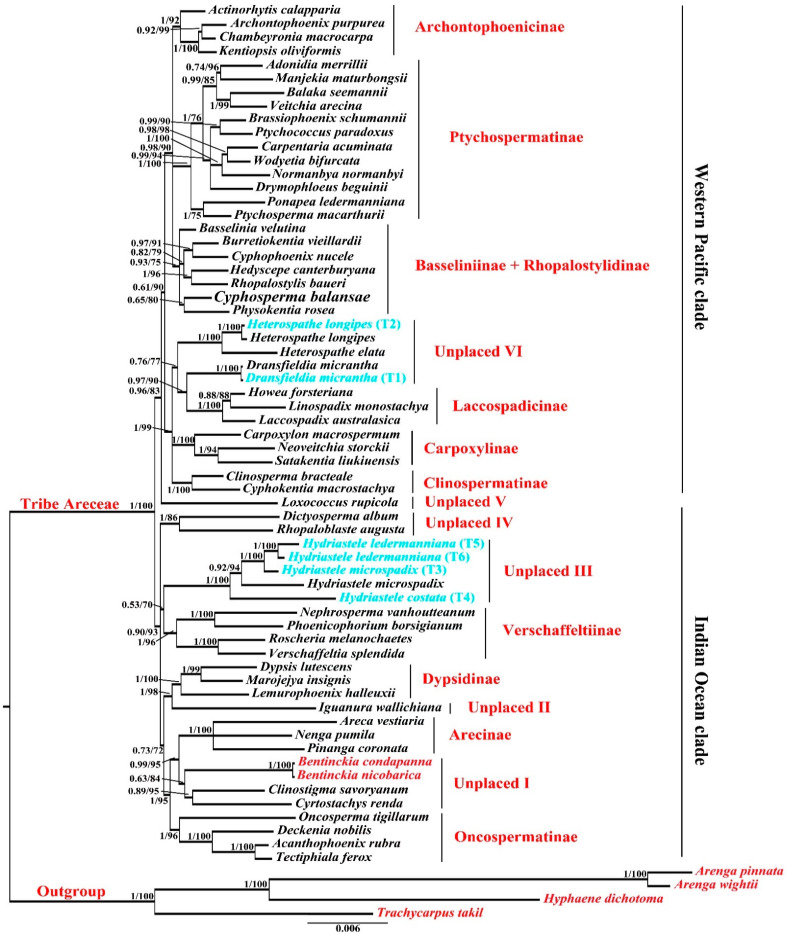
Bayesian phylogeny of the tribe Areceae based on a combined data matrix. BI posterior probability and MI bootstrap values (BI PP/ML BS) are given in front of respected branches. Red colour represents sampled species whereas blue colour represents revised names of species.

The combined matrix of plastid and nuclear loci was used to construct the Maximum Likelihood and Bayesian Inference molecular phylogeny of the tribe Areceae. The aligned sequence dataset of combined nuclear + chloroplast includes 67 genera covered of 7026 characters (Supplementary Material S1). The constructed phylogeny based on the combined dataset resolves nine subtribes with strongly supported clades, including Archontophoenicinae (PP = 1 and BS = 92), Ptychospermatinae (PP = 1 and BS = 100), Laccospadicinae (PP = 1 and BS = 100), Clinospermatinae (PP = 1 and BS = 100), Carpoxylinae (PP = 1 and BS = 100), Verschaffeltiinae (PP = 1 and BS = 96), Dypsidinae (PP = 1 and BS = 100), Arecinae (PP = 1 and BS = 100), and Oncospermatinae (PP = 1 and BS = 100) (Figure 5). However, Basseliniinae and Rhopalostylidinae were clustered in a single group. In addition, both sampled *Bentinckia* species grouped together and formed a clade with other unplaced Areceae.

### 3.3. Ancestral Area Reconstruction

**Figure 6:**
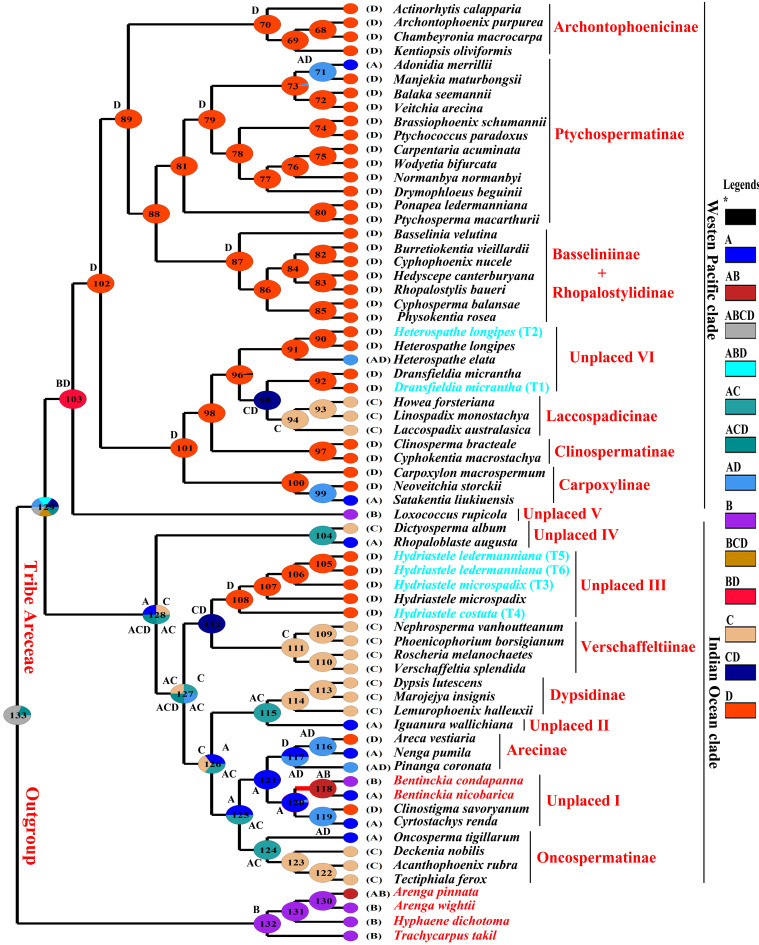
Biogeographic analysis of Areceae based on the Bayesian all compatible groups tree. * (Black colour), ranges with probabilities < 5% are hidden and lumped together and reported as *.

Ancestral area reconstruction of the tribe Areceae was performed using S-DIVA method. The reconstruction based on the combined datasets (Nuclear + Chloroplast) showed a Maximal S-DIVA value of 5099.00 (Figure 6). Node 129 signifies an equal probability of Eurasia (A), India and Sri Lanka (B), Indian Ocean Islands and Madagascar (C), and the Pacific (D) to be an origin of the tribe Areceae. Biogeography analysis showed that Eurasia might be a place of origin of the sampled genus *Bentinckia*. The group of unplaced Areceae containing *Bentinckia*, *Clinostigma*, and *Cyrtostachys* also originated in Eurasia. The other genus, *Iguanura*, originated in Eurasia and the Indian Ocean with 100% probability. The origin of the genus, *Hydriastele*, was found to be in the Indian Ocean and the Pacific. The group of unplaced Areceae IV containing *Dictyosperma* and *Rhopaloblaste* diverged from the rest of the Indian Ocean clade at the Indian Ocean, Eurasia, and the Pacific and distributed into the Indian Ocean. The origin of *Dransfieldia* and *Heterospathe* might be the Pacific and the Indian Ocean and the Pacific, respectively. The genus *Loxococcus* might have originated from India and the Pacific (Figure 6).

## 4. Discussion

The Phylogenetic analysis of Areceae was carried out on nuclear and chloroplast regions. The tribe Areceae is one of the largest and most important tribes from Arecaceae. Several attempts were carried out to find the relationships between the tribe, but it remains poorly understood. In the current study, we have discovered the correct combination of the molecular marker to achieve a better phylogenetic resolution in the tribe Areceae. By using the same dataset, we studied on the phylogenetic placement and evolutionary history of unplaced Areceae members. This study also gives phylogenetic support to the previous systematic revisions carried out in unplaced Areceae. In addition, it will help to resolve the question of unplaced Areceae and future doubts in the tribe Areceae. In addition, this study reports a taxonomic revision and a new cytotype with 2*n* = 30 chromosomes for *Bentinckia*. This study is the first report of 2*n* = 30 chromosomes from the genus *Bentinckia* and the tribe Areceae.

### 4.1. Taxonomic Treatment of Bentinckia

*Bentinckia* Berry ex Roxb., Fl. Ind. 3: 621 (1832).

Type: *Bentinckia condapanna* Berry ex Roxb.

*Keppleria* Mart. ex Endl., Gen. pl. 251 (1837). 

Type: *Keppleria tigillaria* (Jack) Meisn.

Unarmed palms. Leaves terminal, equally pinnate, spathes numerous, two lower short incompletes, upper 2-fid. Spadix interfoliar, branched; flowers minute, monoecious or polygamous, solitary or 3-nate with the intermediate female clustered in spirally arranged form. Pits on the branches, bracts forming a 2-lipped mouth to each pit; bracteoles 2. Male flower sub symmetric, glumaceous, often reduced to ciliate scales; sepals oblong, obtuse, connate below, imbricate; petals longer, connate bellow into a stipes, valvate; stamens 6, anthers versatile; pistillode conical. Female flower ovoid; sepals broad, obtuse, imbricate; petals longer, convolute; staminodes 6. Ovary 3-celled, 1-ovuled; stigmas 3, recurved. Fruit 1.3–1.5 cm in diameter, subspherical. Seeds pendulous from the top of the cavity, sinuately grooved or ridged; albumen equable.

Key to the species of *Bentinckia*

1. Stem slender, up to 10 m tall, flowering branches light pink around pits, ripened fruits deep scarlet; Southern India. *B. condapanna*.

2. Stem robust, up to 20 m tall, flowering branches yellowish white, ripened fruits deep brown; Nicobar Islands. *B. nicobarica*.

*Bentinckia condapanna* Berry in Roxb., Fl. Ind. 3: 621. 1832; Griff., Calcutta J. Nat. Hist. 5 467. 1845; Griffith, Palms Brit. Ind. 160. 1850; Mart., Hist. Nat. Palm. 3: 165, 228. t. 1–39. 1823–1853; Becc. & Hook. f. in Hook. f., Fl. Brit. India 6: 418. 1892; Hook., Fl. Brit. India 6: 418. 1894; Fischer in Gamble. F1. Madras. 1555–1556. 1931; Basu & Chakraverty, Man. Cult. Palms India, 128. 1994; David, Palms Throughout the World, 142. 1995; Renuka & Sreekumar, A field guide to the palms of India, 32–33. 2012.

**Figure 7:**
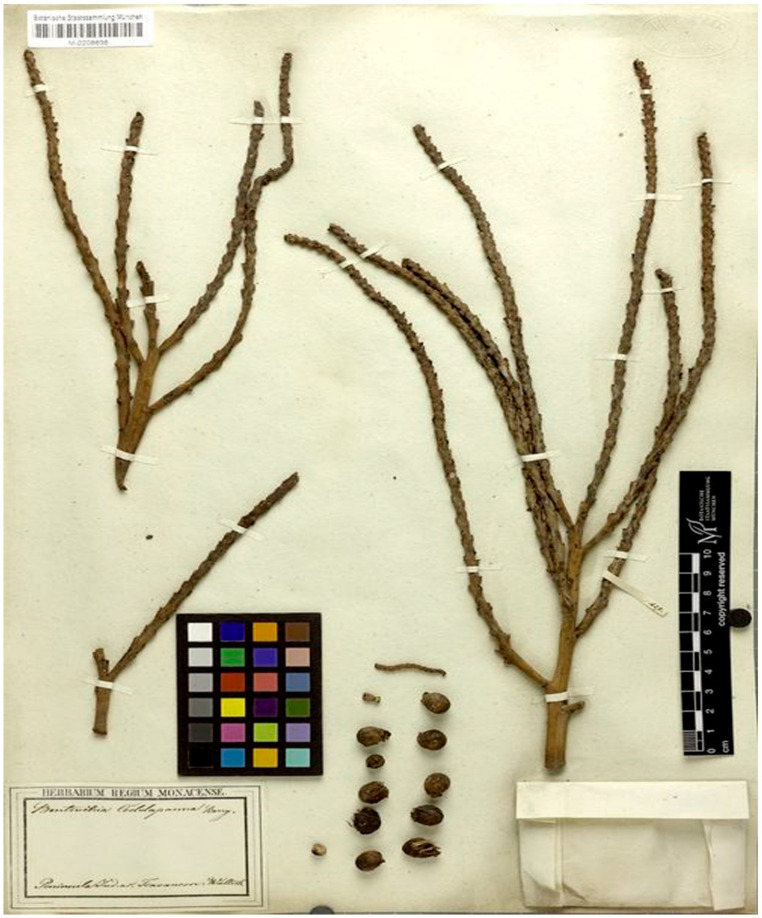
Neotype of *Bentinckia condapanna* Berry ex Roxb. (M0208636). © Botanische Staatssammlung München (M).

Neotype (Designated here): India, Peninsular India or Travancore, s.d., N. Wallich s.n. (M0208636) (Figure 7).

**Figure 8:**
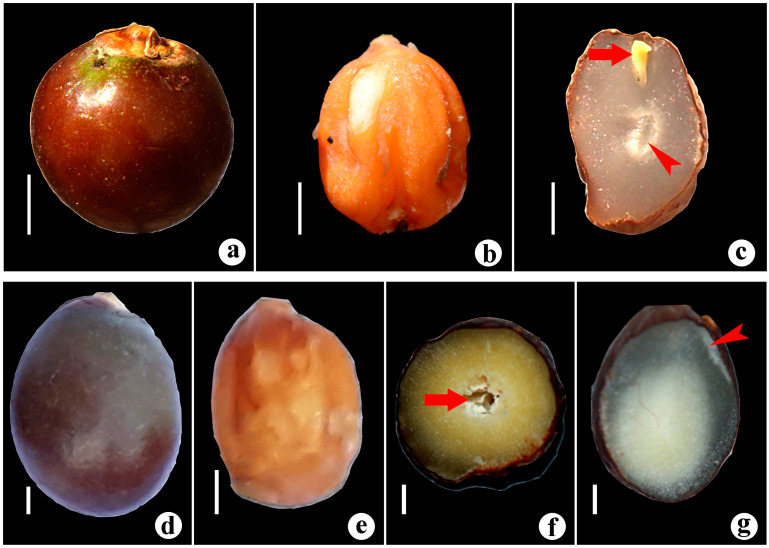
Fruits and seeds in *Bentinckia* species. *B. condapanna* (**a**) fruit, (**b**) seed, and (**c**) cross section of seed. Arrow shows apical embryo and arrowhead horny albumen. Scale bars = 0.2 cm. *B. nicobarica* (**d**) fruit (**e**) seeds, and (**f**,**g**) cross section of seed. Arrow shows ribbed albumen and arrowhead apical embryo. Scale bars = 0.2 cm.

Solitary slender stemmed monoecious palm, stem erect, up to 8 m long, ca. 20 cm diameter near base; crownshaft cylindrical ca. 1 m long. Leaves pinnate, 1–1.5 m long, ascending to spreading in all directions; leaflets linear, acuminate, deep green in colour, up to 80 cm long, to 4 cm broad at middle, duplicately folded near the point of attachment; midnerve conspicuous on upper side bifurcating into long narrow lobes. Inflorescence infrafoliar, decompound; prophyll and peduncular bract large bicarinate, 25–30 cm long, fall off after emergence of flower branches; peduncle flattened, deep green in colour, approximately 4.5 cm long; basal flower branches bracteate, divided into fourth order. Fruit globose to ovoid, bright chocolate coloured when ripe, 1.3–1.5 cm in diameter, seed shining brown, conspicuously grooved adaxially and laterally; endosperm homogenous. Seeds fleshy pendulous in fruit cavity, suspended from the top, grooved and ringed; albumen is horny. Seeds ovate to oblong, conspicuously grooved, convex on one side, ribbed. Embryo is closer to apex, slightly lateral (Figure 8a–c).

Nomenclatural Note: The name *Bentinckia condapanna* Berry ex Roxb. was initially proposed by Berry [9] and was validated by Roxburgh [9] in his *Flora Indica*. In the protologue, Roxburgh [9] stated that Dr. Berry found this plant species in the mountains of Travancore. Berry was a surgeon in the East India Company in Madras and was superintendent of the Company’s Cactus Garden at Marmalong in 1790. It is unclear whether Berry sent the plant to Roxburgh at Calcutta Botanical Garden, and whether it grew well there and Wallich made the specimens or not. We could not locate Berry’s specimens anywhere. However, while searching, we could locate six specimens of *B. condapanna* in M (M0208631, M0208632, M0208633, M0208636, M0208635, M0208634). All these specimens bear a label ‘Peninsula Ind. or. Travancore Wallich’ but are not an original material for the name *Bentinckia condapanna*. Since no original material appears to be extant, the Wallich specimen (M0208636) in M is chosen here as the neotype.

Etymology: The specific epithet ‘condapanna’ was derived from the local terms ‘conda’, used to describe the characteristic casual hairstyle in the local language that resembles opened inflorescence of the palm, and ‘pana’, which designates a colloquial term for palm.

Distribution: India (Kerala and Tamil Nadu).

Habitat: Grows only in the steep slopes of evergreen forests. Locally common on rocky cliffs between 1000 and 1900 m above sea level. 

Local Names: Malayalam (Kantal, Kanthakamugu, Kantha-kamugu, Kanthal, Parapakku, Vareikamuku); Tamil (Kantha Panai, Varei Kamugu, Varukamuvu).

Common Name: Hill Areca nut.

Uses: The palm heart is edible; inflorescences are used in religious ceremonies by the tribal people; the trunk is used for construction purposes; and planted as an ornamental species.

Conservation Status: Vulnerable [3,36].

Note: This species is reported to be rare; distribution in the Western Ghats is restricted to the south Palakkad gap; mainly in the mountains of Agasthymala, Peerumedu, and the Palani Hills [3]. Basu [14] has enlisted this plant under the rare category in his Red Data Book on Indian plants. The World Conservation Monitoring Centre (1996) also considered the species in the rare category. However, a recent field survey assessed the species as Endangered (EN)-A1c, B2a, bi, ii, iii, CI, E. [5].

Specimens examined: INDIA. Kerala: Quilon district, Ponnambala medu, 15 December 1981, C. N. Mohanan 72826 (CAL); Vallakkadavu, Peerumedu, Idukki, 19 October 1996, P. V. Anto 7325 (KFRI); Pullupara, Vallakadav, 20 November 2001, V. B. Sreekumar & V. V. Rangan 7606 (KFRI); Tamil Nadu: Kanyakumari district, Upper kodayar, 7 August 1977, A. N. Henry 49651 (CAL); Salem district, botanical garden, Botanical Survey of India, Yercaud, 10 August 2019, R. N. Mane 144 (SUK).

*Bentinckia nicobarica* (Kurz) Becc. Annales du Jardin Botanique de Buitenzorg 2: 165. 1885; Becc. & Hook. f. in Hook. f. Fl. Brit. India 6:418. 1892; Blatt. Palms Brit. Ind. & Ceyl. 376, t. 67. 1978 (Repr. ed.); Hook., Fl. Brit. India 6: 418. 1894; Basu & Chakraverty, Man. Cult. Palms India, 129. 1994; David, Palms Throughout the World, 142. 1995; Renuka & Sreekumar, A field guide to the palms of India, 34–35. 2012. 

*Orania nicobarica* Kurz, J. Bot. 13: 331 (1875).

**Figure 9:**
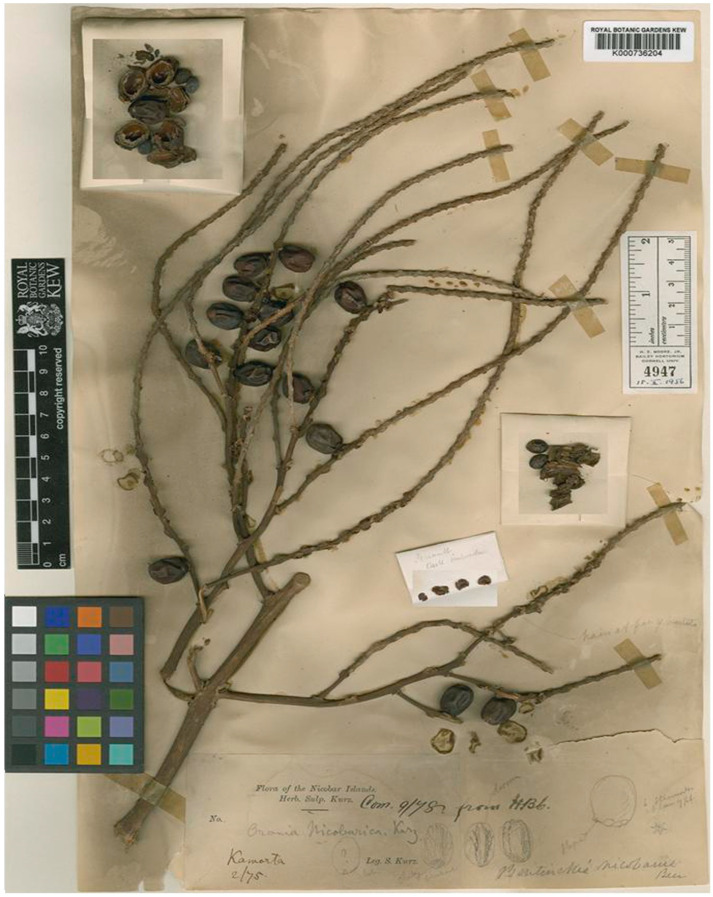
Lectotype of *Orania nicobarica* Kurz (≡*Bentinckia nicobarica* (Kurz) Becc.) (K000736204). © Royal Botanic Gardens, Kew.

Lectotype (designated by Mane and Lekhak, [37]): INDIA, Nicobar Islands: Kamorta, February 1875, W. S. Kurz, s.n. (K000736204) (Figure 9).

Solitary tall palm, stem columnar, distinctly annulate, up to 20 m long, up to 40 cm in diameter near base; crownshaft cylindrical, green, ca. 1 m long. Leaves ascending to arching, approximately 2.5 m long; leaflets closely packed, linear lanceolate, acuminate, alternate to subopposite in adult trees; laterally jointed in younger plants, 50–60 cm long with conspicuous midnerve on upper side; terminal leaflets jointed. Inflorescence infrafoliar, decompound; prophyll and peduncular bracts large, green bicarinate, spatuliform; flower branches greenish yellow; ultimate flower branches slightly inserted at the point of attachment; flowers bracteolate. Fruit subglobose to ellipsoid, deep brown in colour; middle portion fibrous; inner portion brittle; seed ovoid 0.8–0.9 cm long, endosperm white, homogenous. Showing ovoid-oblong seed, ventrally flat, dorsally convex and rugosely ribbed, seed showing apical embryo, cross section of seed showing ribbed albumen (Figure 8d–g).

Nomenclatural Note: The binomial *Bentinckia nicobarica* (Kurz) Becc. was based on basionym *Orania nicobarica* Kurz. In search of type, Mane and Lekhak [37] could locate two specimens of *O. nicobarica* at K (K000736204 and K000736203) and four specimens at CAL. (CAL0000001211, CAL0000001212, CAL0000001213 and CAL0000001214). All specimens serve as syntypes, and Mane and Lekhak [37] designated a specimen from K (K000736204) collected by Kurz from the Nicobar Islands as a lectotype. 

Etymology: The specific epithet ‘nicobarica’ was given after the type locality, i.e. the Nicobar Islands.

Distribution: INDIA (Nicobar Islands). Known only from the Nicobar Islands, being more common in the northern islands according to Kurz [38]. Widely cultivated throughout South East Asia.

Habitat: Lowland evergreen forests at 100–150 m elevation.

Common Names: Bentinck palm, Nicobar palm.

Uses: The tree trunk of this species is used for construction. This species is grown in gardens as an ornamental palm.

Conservation Status: Endangered [39], Critically Endangered [3].

Specimens examined: INDIA, Nicobar Islands: Arong, Car Nicobar, 3 October 2008, E. L. Linto 10724 (KFRI); West Bengal: Acharya Jagadish Chandra Bose Indian Botanic Garden, Kolkata, 5 February 2018, R. N. Mane 110 (SUK).

### 4.2. Cytogenetics

A new cytotype with 2*n* = 30 was reported for *B. condapanna*. In contrast, Sharma [18] reported 2*n* = 32 from the same species. The diploid chromosome ranges from 2*n* = 30 in *B. condapanna* to 2*n* = 32 in *B. nicobarica*. Read [19] reported 2*n* = 32 in the species, and it has been confirmed in the present study; karyotype analysis is also provided. The earlier authors studied chromosomes, and the most common somatic chromosome number was 2*n* = 32 which suggests that the base number (*x*) for the genus is 16. The intrachromosomal index (M_CA_) is due to the centromeric position while the interchromosomal index (CV_CL_) depicts heterogeneity among chromosome sizes in a complement. Higher values of M_CA_ and CV_CL_ for *B. nicobarica* indicate more asymmetry in its karyotype. The *B. nicobarica* karyotype shows two types of chromosomes, m and sm, whereas in *B. condapanna*, only m type chromosomes are present. In addition, the high R ratio in *B. nicobarica* reflects more heterogeneity in its chromosome complement.

The sequence of basic chromosomes in palms (*n* = 13–18) is known as the dysploid series [1]. The chromosomes in Arecaceae show a great variability in length and chromosome number (*Voanioala gerardii* 2*n* = 550, 596, 606) [1]. The family Arecaceae is large and morphologically diverse. Metacentric to submetacentric (m to sm) chromosomes are dominant in Areceae [1]. Our study also showed that metacentric and submetacentric chromosomes are present in *Bentinckia*.

### 4.3. Molecular Phylogeny of Areceae 

The palm family is considered among the oldest and most well-studied families. Arecoideae and Areceae are the largest subfamily and tribe from Arecaceae, respectively. Many studies have reported phylogenetic analysis of Areceae; however, the relationship within the tribe is still ambiguous. Several reports strongly support the monophyly of Areceae [40,41,42,43,44] while others recover it with less support [45,46,47,48]. In addition, a few other studies also reported the phylogenetic relationship of Areceae [49,50,51,52], but all of them were unable to report a well-resolved molecular phylogeny of Areceae. Many authors reported phylogeny based on single plastid or nuclear DNA (PRK and RPB2) regions. However, the role of low-copy nuclear and chloroplast DNA becomes very important while studying the phylogeny of palm. Therefore, the right combination of molecular markers is inevitable for a well-resolved phylogeny [20]. Areceae contains 11 subtribes, 61 genera (10 unplaced), and 660 species, even though the phylogenetic positions of several genera are not well understood. Here, we are reporting a well-resolved phylogeny of Areceae built using the appropriate combination of chloroplast and nuclear regions. Based on this phylogeny, it is clear that Areceae is divided into two main groups: the Western Pacific clade and the Indian Ocean clade. The Indian Ocean clade consists of four subtribes: Oncospermatinae, Arecinae, Dypsidinae, and Verschaffeltiinae while the Western Pacific clade consists of seven subtribes: Archontophoenicinae, Ptychospermatinae, Basseliniinae, Rhopalostylidinae, Laccospadicinae, Clinospermatinae, and Carpoxylinae. Based on PRK and RPB2, Norup et al. [43] found that the Western Pacific and Indian Ocean clades are monophyletic. Nevertheless, the interrelationship within the members of the subtribes remained unresolved on account of polytomy.

#### Unplaced Areceae

The ten unplaced members of Areceae are divided into six groups and they are distributed among the Western Pacific and the Indian Ocean clade. The sampled Indian endemic *Bentinckia* groups together and forms a clade with other unplaced *Clinostigma* and *Cyrostachys*. This clade of unplaced Areceae shows a sister relationship with the subtribe Arecinae. This relationship was recovered in both the supertree and the supermatrix analysis conducted by Baker et al. [49]. Supertree and supermatrix analyses were carried out using the same 16 partitions; however, the phylogenetic relationships within Areceae are in contrast with each other. *Iguanura wallichiana* shows a sister relationship with subtribe Dypsidinae with strong support. The clade of unplaced *Hydriastele* stands confidently showing a sister relation with the subtribe Verschaffeltiinae. Additionally, Petoe et al. [53] recognized both the former species *Gronophyllum chaunostachys* (Burret) H.E.Moore and *Hydriastele chaunostachys* (Burret) W.J.Baker & Loo as *Hydriastele ledermanniana* (Becc.) W.J.Baker & Loo [54], and these two former species group together and show close relationships with other *Hydriastele* species in our phylogeny. Baker and Loo [55] synonymized *Gulubia macrospadix* (Burret) H.E.Moore as *Hydriastele microspadix* (Burret) W.J.Baker & Loo [54], which is also shown by our findings, as *G. macrospadix* nests within other accessions of *Hydriastele microspadix*. Similarly, *Gulubia costata* (Becc.) Becc., a synonym of *Hydriastele costata* F.M.Bailey [55], shows a close relationship with other *Hydriastele* species. Therefore, our phylogeny of Areceae supports all the revised circumscriptions of *Gronophyllum*, *Gulubia*, and *Hydriastele*. The paraphyletic clade of unplaced *Dictyosperma* and *Rhopaloblaste* shows a close relationship with the remaining species of the Indian Ocean clade. Baker et al. [44] also recovered this clade representing *Dictyosperma* and *Rhopaloblaste*. In addition, this study resolved the phylogenetic position of a few of the subtribes but with very low support. Most of them resolved as a polytomy.

In the Western Pacific clade, the unplaced member of Areceae *Loxococcus* stands confidently as a sister to the western Pacific clade. Other unplaced members, *Dransfieldia* and *Heterospathe*, show a close relation with the subtribe Laccospadicinae. *Dransfieldia* shows a sister relationship with the subtribe Laccospadicinae and is placed in the same clade while *Heterospathe* stands as a sister to that clade. In addition, Baker et al. [56] revised the former species *Ptychosperma micranthum* Becc. as *Dransfieldia micrantha* (Becc.) W.J.Baker & Zona [54] and both species show a close relationship in our proposed phylogeny. Further, Norup [57] revised former species *Alsmithia longipes* H.E.Moore as *Heterospathe longipes* (H.E.Moore) Norup [54], and our phylogeny supports this. Loo et al. [42] also showed molecular support for the inclusion of some species of *Gronophyllum* and *Gulubia* in *Hydriastele.* Our phylogeny supports this inclusion. In addition, it also provides molecular support to the revisions carried out in *Ptychosperma*, *Alsmithia*, and *Hydriastele* species concerned with unplaced Areceae. 

### 4.4. Ancestral Area Reconstruction of Areceae

Studying evolutionary history based on molecular phylogeny is obligatory to understand precise biogeographical evolution [58]. To date, no robust biogeographical work has been carried out on the tribe Areceae. Previous reports [33,34] have studied the biogeography of the palm; however, the relationship within Areceae, as well as Arecaceae, was not resolved [20]. The main focus of those studies was the family or subfamily-level relationship. Our S-DIVA analysis suggests Eurasia (A), India and Sri Lanka (B), Indian Ocean Islands and Madagascar (C), and the Pacific (D) may have been the centre of origin of the tribe Areceae. The unplaced members of Areceae, including *Bentinckia*, *Clinostigma*, and *Cyrtostachys*, show Eurasia might be the centre of origin of this group. *Bentinckia* diverged from *Clinostigma* and *Cyrtostachys* in Eurasia and was distributed in Indian territory. *Bentinckia condapanna* is spread in Kerala and Tamil Nadu while *Bentinckia nicobarica* remains endemic to the Nicobar Islands. Baker and Couvreur [33] reported a contrasting result to our study. These authors showed *Cyrtostachys* diverged from *Bentinckia* and *Clinostigma. Iguanura* diverged from the subtribe Dypsidine in Eurasia and the Indian Ocean while Baker and Couvreur [33] reported the diversion of *Iguanura* from the rest of Areceae. Our biogeography analysis shows that *Hydriastele* originated in the Indian Ocean and the Pacific and diverged from the subtribe Verschaffeltiinae. However, the previous study reported that *Hydriastele* diverged from the subtribe Oncospermatinae [33,34]. *Dictyosperma* and *Rhopaloblaste* might have originated in Eurasia, the Indian Ocean, and the Pacific and then diverged from the rest of the Indian Ocean clade. The genus *Loxococcus* diverged from the Western Pacific clade in India and the Pacific. The origin of *Dransfieldia* and *Heterospathe* might be the Pacific and the Indian Ocean and the Pacific, respectively. *Heterospathe*, *Dransfieldia*, and the subtribe Laccospadicinae diverged like *Heterospathe*—(*Dransfieldia*—Laccospadicinae). All the results of the biogeography reported by Baker and Couvreur [33] were based on a supertree of the palm of Baker et al. [49]. This study could not resolve the relationship within the tribe Areceae as the relationship within the tribe was not well understood at that time. 

## 5. Conclusions

The present study reports a revision of *Bentinckia* and assigns a neotype for *B. condapanna*. In addition, it documents a new cytotype for *B. condapanna* with 2*n* = 30 chromosomes, which is the first different chromosome number reported from the genus *Bentinckia* and the tribe Areceae. The genus *Bentinckia* needs to be conserved because both species fall in a threatened category. As per the IUCN, *B. condapanna* is considered in the vulnerable (A1c, B2a ver 3.1) [5] category, and *B. nicobarica* is listed in the endangered (C2a ver 2.3) [39] category. This study provides the first well-resolved phylogeny of Areceae built using the appropriate combination of chloroplast and nuclear regions that supports all previous systematic revisions from unplaced Areceae. Moreover, molecular phylogeny and biogeographic analysis give the phylogenetic position and evolutionary history of all unplaced Areceae concerning closely related subtribes. However, to assign respective subtribes to unplaced genera, a strong morphological support is necessary. In addition, for precise evolutionary history, more sampling from Areceae is necessary. Nevertheless, this study confirms the phylogenetic placement and evolutionary history of *B. condapanna* and *B. nicobarica*. Phylogenetic analysis revealed that both species of *Bentinckia* form a clade with the other unplaced members, *Clinostigma* and *Cyrostachys*, and together they show a sister relationship with the subtribe Arecinae. Biogeography analysis shows *Bentinckia* might have originated in Eurasia and India.

## Figures and Tables

**Table 1:** Comparative karyotypes of *Bentinckia condapanna* and *B. nicobarica*.

	*B. condapanna*	*B. nicobarica*
2*n*	30	32
Total haploid chromosome length (THCL ± SE (µm))	34.04 ± 0.59	25.83 ± 0.40
Haploid karyotype formula	15m	9m + 7sm
Coefficient of Variation of Chromosome Length (CV_CL_)	18.18	27.17
Coefficient of Variation of Centromeric Index (CV_CI_)	8.05	11.17
Mean Centromeric Asymmetry (M_CA_)	14.05	22.02
Shortest chromosome (S ± SE (µm))	1.48 ± 0.34	0.91 ± 0.25
Longest chromosome (L ± SE (µm))	3.00 ± 0.25	2.30 ± 0.16
Mean chromosome length (MCL ± SE (µm))	1.62 ± 0.59	1.61 ± 0.11
Longest to shortest chromosome ratio (R)	2.03	2.52
Stebbins category (St)	4B	3B

## Data Availability

Data supporting the findings of this study are available within the article and its Supplementary Materials.

## Supplementary Materials

The following supporting information can be downloaded at: https://www.mdpi.com/article/10.3390/biology12020233/s1, Supplementary Material S1: Aligned combined sequence data (PRK + RPB2 + *acc*D + *rpo*C1 + *rbc*L + *rps*16 + *trn*L-F + *mat*K + *ndh*F) matrix. Supplementary Table S1: Voucher information and GenBank numbers (PRK, RPB2, *rbc*L, *rps*16, *mat*K, *ndh*F, *rpo*C1 *acc*D, and *trn*L-F) for all accessions used in this study. The sequences generated in this study are marked with * and — represent missing sequences.
