# Supplementary material for: Cytogenetics, Typification, Molecular Phylogeny and Biogeography of Bentinckia (Arecoideae, Arecaceae), an Unplaced Indian Endemic Palm from Areceae
Source: Biology (Basel). 2023 Feb 1;12(2):233. doi: 10.3390/biology12020233 (PMC9952971; doi:10.3390/biology12020233)
Supplement: Supplementary file 1 [file biology-12-00233-s001.zip › Supplimentary Table S1.pdf]

Supplementary Table S1. Voucher information and GenBank numbers (PRK, RPB2, *rbc*L, *rps*16, *mat*K, *ndh*F, *rpo*C1 *acc*D, and *trn*L-F) for all accessions used in this study. The sequences generated in this study are marked with * and – represent missing sequences.

Name of taxa	GenBank accession numbers								
	**PRK**	**RPB2**	***rbc*L**	***rps*16**	***mat*K**	***ndh*F**	***rpo*C1**	***acc*D**	***trn*L-F**
Actinorhytis calapparia	AJ831223	AJ830024	MG437684	MG647500	HG969979	MG647241	MG438196	MG437943	HG969945
Acanthophoenix rubra	AF453329	AJ830020	AJ829844	AM116836	AM114691	MG647281	MG438236	-	AM113679
Adonidia merrillii	KJ598276	AJ830193	MG437730	MG647546	MK704941	MG647285	MG438239	MG437986	AB817688
Archontophoenix purpurea	AJ831227	AJ830028	MG437685	MG647501	HG969974	MG647242	MG438197	MG437944	HG969940
Areca vestiaria	LT671885	LT671910	AY012497	AY044584	MK705054	AY044535	-	-	-
Arenga pinnata	OP009482	OP009494	ON981421	ON981433	OP009506	ON996963	-	-	-
Arenga wightii	OP009484	OP009496	ON981423	ON981435	OP009508	ON996965	-	-	-
Balaka seemannii	JF833372	JF833395	AJ404814	AJ240896	AM114695	MG647288	MG438242	MG437989	AJ241305
Basselinia velutina	-	-	MG437693	MG647509	AM114667	MG647250	MG438205	MG437952	AM113662
Bentinckia condapanna	OQ032520*	OQ032522*	OQ032524*	OQ032526*	OQ032528*	-	-	-	-
Bentinckia nicobarica	OQ032521*	OQ032523*	OQ032525*	OQ032527*	OQ032529*	OQ032530*	MG438256	MG438003	AM113687
Brassiophoenix schumannii	KJ598275	-	MG437739	AJ240897	AM114699	MG647294	MG438248	MG437995	AJ241306
Burretiokentia vieillardii	AJ831243	AJ830039	MG437694	MG647510	-	MG647251	MG438206	MG437953	-
Carpoxylon macrospermum	AF453337	AJ830055	MG437701	MG647517	AM114673	MG647258	MG438213	MG437960	AM113667
Carpentaria acuminata	AJ831259	AJ830196	MG437735	HG969818	HG969984	MG647290	MG438244	MG437991	HG969950
Chambeyronia macrocarpa	AJ831260	AJ830056	MG437687	MG647503	HG969962	AY044536	MG438199	MG437946	HG969931
Clinostigma savoryanum	AJ831263	AJ830059	MG437748	AF449148	LC680314	MG647303	MG438257	MG438004	AM113688
Clinosperma bracteale	AJ831261	AJ830057	MG437706	MG647522	AM114680	MG647263	MG438217	MG437964	AM113674
Cyrtostachys renda	AF453341	AJ830062	MG437749	AJ404940	AM114707	MG647304	MG438258	MG438005	-
Cyphophoenix nucele	AJ831266	AJ830061	AJ404821	AJ240900	AM114669	MG647253	MG438208	MG437955	AJ241309
Cyphosperma balansae	-	AY543098	MG437698	MG647514	HG969988	MG647255	MG438210	MG437957	HG969954
Cyphokentia macrostachya	AJ831264	AJ830060	MG437704	MG647520	-	MG647261	MG438215	MG437962	AM113670
Deckenia nobilis	AF453342	AJ830063	MG437725	MG647541	-	MG647280	MG438235	MG437983	-
Dictyosperma album	AF453343	AJ830064	MG437750	AM116846	AM114708	MG647305	MG438259	MG438006.	AM113689
Dransfieldia micrantha	AJ831326	AJ830139	MG437751	AM116847	AM114709	MG647306	MG438260	MG438007	AM113690
Dransfieldia micrantha (T1)	AJ831326	AJ830139	AJ829920	-	-	-	MG438260	MG438007	AM113690
Drymophloeus beguinii	KJ598261	AJ830197	AY012494	AY044586	KJ598356	AY044537	-	-	-
Dypsis lutescens	AF453346	AJ830078	MG437709	MG647525	AM114681	EU004903	MG438220	MG437967	-

Hedyscepe canterburyana		AJ971833	MG437742	AJ404938	KJ598380	MG647297	MG438251	MG437998	-
Heterospathe elata	AF453350	AJ830085	MG437752	EF605590	AM114710	MG647307	MG438261	MG438008	AM113691
Heterospathe longipes	AM260638	AM260637	MG437753	MG647569	HG969987	MG647308	MG438262	MG438009	HG969953
Heterospathe longipes (T2)	AJ831226	AJ830027	AJ829850	-	-	-	-	-	-
Howea forsteriana	AJ971828	MK102320	MG437722	MG647538	AM889716	MG647277	MG438232	MG437980	-
Hydriastele costata (T4)	AY348926	AY543128	-	-	-	-	-	-	-
Hydriastele microspadix	AY348935	AY546246	MG437754	AJ404943	AM114712	MG647309	MG438263	MG438010	-
Hydriastele ledermanniana (T5)	AF453349	AY543118	AJ404816	AJ240898	-	-	-	-	AJ241307
Hydriastele ledermanniana (T6)		AJ833635	MG437755	MG647571	AM114713	MG647310	MG438264	MG438011	-
Hydriastele macrospadix (T3)	AY348929	AY543133	-	-	-	-	-	-	-
Hyphaene dichotoma	OP009488	OP009500	ON981427	ON981439	-	-	-	-	-
Iguanura wallichiana	-	AY543099	MG437756	AJ404946	AM114714	MG647311	MG438265	MG438012	-
Kentiopsis oliviformis	AF453353	AY543101	MG437688	MG647504	HG969965	MG647245	MG438200	MG437947	HG969932
Laccospadix australasica	AJ831301	AJ830109	AJ404812	AJ240895	AM114689	MG647278	MG438233	MG437981	AJ241304
Lemurophoenix halleuxii	AF453354	AM260636	AJ404801	AJ404935	AM114682	MG647267	MG438221	MG437968	-
Linospadix monostachya	-	EF491151	AJ404811	EF605586	AM114688	DQ273112	MG438231	MG437979	AB522466
Loxococcus rupicola	AY348943	AY543152	MG437757	AM116851	KJ598377	MG647312	MG438266	MG438013	AM113694
Manjekia maturbongsii	JF833370	JF833393	MG437731	MG647547	KJ598374	MG647286	MG438240	MG437987	-
Marojejya insignis	AJ831307	AJ830122	AM110230	AM116832	AM114684	MG647270	MG438224	MG437971	AM113675
Nenga pumila	AF453361	AY543155	AJ404818	AJ404944	MK705052	MG647247	MG438202	MG437949	-
Neoveitchia storckii	AJ831319	AJ830130	MG437703	MG647519	AM114675	MG647260	-	-	AM113669
Nephrosperma vanhoutteanum	AF453362	AJ830131	AJ829889	MG647559	MK705031	MG647298	MG438252	MG437999	-
Normanbya normanbyi	FJ032019	FJ200372	MG437738	MG647554	KJ598368	MG647293	MG438247	MG437994	-
Oncosperma tigillarum	-	MK102338	AY012505	MG647540	AM114690	AF453474	MG438234	MG437982	HG969949
Pinanga coronata	AY348944	AY543156	MG437691	MG647507	MK705040	MG647248	MG438203	MG437950	-
Physokentia rosea	AJ831322	AJ830138	AJ829896	AM116822	AM114671	MG647257	MG438212	MG437959	AM113665
Phoenicophorium borsigianum	AF453368	AJ830136	AJ829893	AM116843	AM114703	HG969754	MG438253	MG438000	HG969952
Ponapea ledermanniana	KJ598277	AJ830199	MG437729	MG647545	KJ598372	MG647284	MG438238	MG437985	AM113682
Ptychosperma macarthurii	KJ598253	AJ830201	MG437728	MG647544	MK704980	MG647283	MG438237	MG437984	AM113681
Ptychococcus paradoxus	AJ831324	AJ830200	AJ829906	AM116842	AM114700	MG647295	MG438249	MG437996	AM113685
Rhopaloblaste augusta	AF453373	AY543107	AM110244	AM116852	AM114717	MG647313	MG438267	MG438014	AM113695
Rhopalostylis baueri	-	AJ830145	MG437741	HG969819	HG969985	MG647296	MG438250	MG437997	HG969951
Roscheria melanochaetes	AF453374	AJ830140	AJ404822	AJ404947	AM114704	MG647300	MG438254	MG438001	-

Satakentia liukiuensis	AF453376	AJ830146	MG437702	MG647518	AM114674	MG647259	MG438214	MG437961	AM113668
Tectiphiala ferox	AF453380	AJ830148	MG437727	MG647543	AM114692	MG647282	-	-	AM113680
Trachycarpus takil	OP009491	OP009503	ON981430	ON981442	OP009512	ON996968	-	-	-
Veitchia arecina	JF833388	JF833410	AJ404813	AJ404942	AM114696	MG647289	MG438243	MG437990	-
Verschaffeltia splendida	AF453381	AJ830150	AJ829916	EF605588	MK705030	MG647301	MG438255	MG438002	-
Wodyetia bifurcata	KJ598260	AJ830206	MG437736	AM116841	AM114698	MG647291	MG438245	MG437992	AM113684
